# Structural and Functional Neuroimaging Studies of Vascular Risk Factors in the Alzheimer's Continuum: A Systematic Literature Review

**DOI:** 10.1002/alz70856_102870

**Published:** 2025-12-25

**Authors:** Srijan Konwar, Riccardo Manca, Matteo De Marco, Annalena Venneri

**Affiliations:** ^1^ Brunel University London, Uxbridge, United Kingdom; ^2^ University of Parma, Parma, Italy; ^3^ Brunel University London, Uxbridge, Middlesex, United Kingdom; ^4^ Brunel University London, Uxbridge, UB8 3PH, United Kingdom; ^5^ Brunel University London, London, United Kingdom; ^6^ University of Parma, Parma, Italy, Italy

## Abstract

**Background:**

Alzheimer's Disease (AD) tends to co‐exists with other pathologies, with vascular pathology being the most common type. It is well known that the presence of comorbidities results in differential disease trajectories and heterogeneity in clinical phenotypes. The aim of this systematic literature review was to examine the structural and functional neuroimaging patterns of vascular comorbidity in the AD spectrum.

**Method:**

We used the Preferred Reporting Items for Systematic Reviews and Meta‐Analyses (PRISMA) systematic review guidelines to search for articles in databases such as PubMed, Scopus and Web of Science. Selection of participants included younger, middle ‐aged and older adults and only studies of patients in the AD continuum were included. The initial selection included 1435 articles and after a thorough screening based of a series of defined inclusion and exclusion criteria, 67 papers were selected for structural imaging, and 49 papers for functional imaging.

**Result:**

The general findings from these studies were that participants with vascular comorbidity had widespread cortical thinning, volumetric loss and poor white matter integrity, bilaterally, across AD‐susceptible regions in the temporal and parietal lobes but also in frontal and occipital regions. Interestingly, cognitively unimpaired older adults with vascular co‐pathology showed greater functional connectivity in the regions of the sensorimotor and visual networks. This was supported by corresponding evidence of higher thickness in the postcentral gyrus and larger volumes in the deep subcortical regions.

**Conclusion:**

Findings from this systematic literature review reveal that the effect of vascular burden is not uniform and region specific. Rather, it tends to have an effect at the macro level, affecting both AD‐susceptible region, regions of the sensorimotor and visual networks, as well as deeper subcortical regions such as the thalamus, brainstem and cerebellum. Two distinct imaging patterns were observed between cognitive impaired and unimpaired individuals: one is that of widespread cortical thinning, volume loss and weaker functional connectivity between brain regions in impaired individuals; in cognitively unimpaired individuals an opposite pattern was instead detected, with greater regional cortical thickness, larger volumes and stronger functional connectivity.

